# Parliament and the Profession

**Published:** 1918-02-23

**Authors:** 


### Parliament and the Profession.

### National Health Insurance.

The Royal Assent has been given to the National Health Insurance Act, 1918.

### Medical Services in Palestine.

Mr. Macpherson informed Major Newman that he was unaware of any breakdown in the medical or commissariat arrangements during the earlier stages of the British advance from Gaza to Jerusalem. In view of the rapidity of the movement, difficulties naturally increased. There was an increase of intestinal disorders in the force due to the great strain undergone by the troops during the advance, and the coldness of the nights.

### Poisonous Gas: Red Cross Appeal.

Mr. Denman asked the Secretary of State for Foreign Affairs whether he has taken any steps in reply to the Red Cross appeal for the discontinuance of the use of poisonous gas in this war; and whether he is prepared to enter into an agreement on the subject with the other belligerent States. Lord R. Cecil said that he had not yet received the appeal from the Red Cross. His Majesty's Government propose to consult with their Allies as to the action to be taken conjointly in view of this appeal, but he could make no statement on the subject for the present.

☞ Previous articles appeared Feb. 24, March 10, 24, 31, April 14 and 21, May 5, 12, and 19, June 9 and 16, Sept. 1 and 29.

